# The Surgery of Military Spinal Injuries

**Published:** 1917-04-14

**Authors:** 


### THE SURGERY OF MILITARY SPINAL INJURIES.

### Some Indications for its Employment.

The operative treatment of gunshot wounds involving the spine and spinal cord is not by any means new; various surgeons have applied the principles of modern surgery to the special problems which wounds of this region present, with considerable measure of success, but never before this war has any single operator been able to accumulate the ripe experience which many military surgeons now possess. In a recent paper,* Lieut.-Colonel Hull embodies his own views as to the proper indications for surgery in dealing with gunshot wounds of the spine. Coming from an authority of such wide experience and acknowledged eminence, these suggestions for treatment are bound to have a high value for those numerous surgeons in charge of military beds who may at any moment be faced by a case of spinal injury but have not encountered many cases of that sort in the past.

To begin with, two points are emphasised: one is the importance of differentiating between hopeless cases and those likely to benefit by operation; the other the vital need to avoid delay. The earlier circumstances allow the case to be operated on, says Colonel Hull, the better will be the results. The fact that the incision has to be planned through septic tissues should not be taken as a reason for delay.

Indications for declining to operate are total loss of sensation and of voluntary movement in the parts below the spinal-cord lesion. But if, after having been in such a condition, the patient recovers some power of movement or some degree of sensation, operation is justifiable. It is also right to operate when a foreign body is present and has been localised in some accessible situation. Severe pain, even in the absence of other indications, may render operative interference justifiable.

### The Diagnosis of Cauda Equina Lesions.

Injuries to the cauda equina have a more hopeful outlook than those of the spinal cord itself, in which connection it is well to remember that the lumbar segments of the spinal cord do not lie within the corresponding vertebræ, but are placed much higher up: that is, the termination of the spinal cord lies at the level of the lower border of the first lumbar vertebra, and below that level there are only the nerves of the cauda equina within the spinal theca. Pain and hyperæsthesia are evidence of injury to the nerve roots rather than to the cord; and abolition of the deep reflexes has the same significance. A rapid segmental increase of symptoms points to a lesion of the spinal cord; a slow increase to one of the cauda equina.

Great stress is laid upon the importance of local anæsthesia in the operative treatment of these cases, in order to minimise shock, hæmorrhage, and chest complications. A long incision is rightly laid down as one of the essentials for successful laminectomy. Another practical point is the administration of urotropine by the mouth as soon after the injury as possible, to reduce the ever-present danger of urinary infection. The most careful and aseptic catheterisation is, of course, necessary at the same time; and lavage of the bladder with antiseptic solutions is also advantageous. The presence of a missile together with severe pain is an indication for immediate operation. Given an operable lesion, an expert operator, the absence of long delay, and the accurate localisation of foreign bodies, the prognosis is not so gloomy, says Colonel Hull, as past experience teaches.

### The Importance of Drainage of the Bladder.

Several surgeons are very emphatic on the need for supra-pubic drainage of the bladder as a routine part of the operative treatment of these cases, with a view to avoiding the otherwise almost certain occurrence of septic cystitis. Mr. Thomson Walker has lately laid considerable stress on this point, which is well worth the attention of practical surgeons. The writer would even go so far as to say definitely, from personal experience which, though limited, is very striking, that a surgeon is not justified in undertaking laminectomy for gunshot wound unless he is prepared to supplement it by supra-pubic drainage of the bladder.

* *Journal of the Royal Army Medical Corps*, January 1917; p. 66.

